# Stretching of Sciatic, Digital, and Infraorbital Nerves

**Published:** 1880-06

**Authors:** 


### STRETCHING OF SCIATIC, DIGITAL, AND INFRAORBITAL NERVES.

For the following notes we are indebted to Mr. R. Purdie, M. B., C. M.

Case 1. *Stretching of the Sciatic Nerve.*—M. F——, a miner, was admitted in the month of August of last year. He had suffered for several months from sciatica. In consequence of want of rest and exhaustion, caused by the intense paroxysms of pain, the man had become emaciated and debilitated and quite unfit for his occupation.

Before nerve-stretching was used other methods were tried as well as tonic treatment; local means, such as acupuncture of the great sciatic along different parts of its course. The use of galvanism, and lastly the use of galvano-puncture of the sciatic nerve; but none of these remedies produced more than temporary relief.

On the 3d of September, the patient having been brought under the influence of chloroform, an incision one inch and three-quarters in length was made, from the margin of the gluteus maximus along the outer margin of the biceps; the fascia was opened, and the nerve was readily exposed and hooked over the finger. It was then forcibly stretched, while the foot was held somewhat fixed by an assistant. The stretching was continued until the nerve was loosened, and a sense of yielding obtained. The wound was then lightly dressed, and healed by the first intention.

Since leaving the hospital (now more than a year since) occasional slight twichings have been felt in damp weather, but nothing compared to the intense pain suffered before the operation.

Case 2. *Stretching of the Digital Nerves.*—The patient, D. H——, suffered from whitlow on the dorsal surface of terminal phalanz of the forefinger seven years ago; after the pus had been evacuated stiffness of the joint at the phalanx gradually set in, accompanied by a severe pain of a paroxysmal character, occurring most frequently at night, and lasting from about a quarter to one hour. This pain began at the tip of the finger and shot upwards as far as the proximal phalanx. All feasible remedies were tried with no satisfactory result. On the 30th June, the patient having been put under chloroform and an elastic band tied tightly round the finger, Mr. Spence, by longitudinal incisions, exposed the digital nerves. He then introduced a blunt hook under each nerve, and stretched these forcibly. The patient was able to walk home on the same day.

The wounds soon healed, and there has been complete freedom from pain since.

Case 3. *Stretching of the infra-orbital Nerve for Epileptiform Neuralgia.*—The patient, C. D——, was admitted into the infirmary on the 3rd of April last, suffering from severe neuralgic attacks on the left side of the nose, left cheek, and eyebrow, and shooting up over the forehead. His first attack was in December, 1876, continuing more or less for a month. After a period of four months he was again attacked. On that occasion some relief was obtained by the extraction of some of the teeth of the upper jaw. Ultimately the pain became almost incessant, being liable to be set up by the slightest touch or movement, as in swallowing, etc., or by draughts of cold air. The attacks were most severe about midnight, and were usually preceded by a cold shiver running down the spine.

On May 22nd, the patient being put under chloroform, Mr. Spence exposed the infra-orbital nerve by a transverse incision. He then introduced a blunt hook under the nerve, and fixing the upper lip with the left hand, stretched the nerve with considerable force.

On the fifth day after the operation a slight return of the pain took place, and, as the incision had not united, Mr. Spence again stretched the nerve.

The wounds healed kindly, and there has been no return of the pain since.—*London Lancet.*

